# Absolute Measurements of Macrophage Migration Inhibitory Factor and Interleukin-1-β mRNA Levels Accurately Predict Treatment Response in Depressed Patients

**DOI:** 10.1093/ijnp/pyw045

**Published:** 2016-05-11

**Authors:** Annamaria Cattaneo, Clarissa Ferrari, Rudolf Uher, Luisella Bocchio-Chiavetto, Marco Andrea Riva, the MRC ImmunoPsychiatry Consortium, Carmine M. Pariante

**Affiliations:** Stress, Psychiatry and Immunology Laboratory, Department of Psychological Medicine, Institute of Psychiatry, Psychology and Neuroscience, King’s College London, London, United Kingdom (Drs Cattaneo and Pariante); Biological Psychiatry Unit, IRCCS Fatebenefratelli Institute, Brescia, Italy (Dr Cattaneo); Statistical Service, IRCCS Fatebenefratelli Institute, Brescia, Italy (Dr Ferrari); Genetic Unit, IRCCS Fatebenefratelli Institute Brescia, Italy and Faculty of Psychology, eCampus University, Novedrate, Como, Italy (Dr Bocchio-Chiavetto); Department of Psychiatry, Dalhousie University, Halifax, Nova Scotia, Canada (Dr Uher); The Social, Genetic, and Developmental Psychiatry Centre, Institute of Psychiatry, Psychology and Neuroscience, King’s College London, London, United Kingdom (Dr Uher); Department of Pharmacological and Biomolecular Sciences, University of Milan, Milan, Italy (Dr Riva).

**Keywords:** cytokine absolute blood levels, treatment response, predictors, personalized medicine

## Abstract

**Background:**

Increased levels of inflammation have been associated with a poorer response to antidepressants in several clinical samples, but these findings have had been limited by low reproducibility of biomarker assays across laboratories, difficulty in predicting response probability on an individual basis, and unclear molecular mechanisms.

**Methods:**

Here we measured *absolute* mRNA values (a reliable quantitation of number of molecules) of Macrophage Migration Inhibitory Factor and interleukin-1β in a previously published sample from a randomized controlled trial comparing escitalopram vs nortriptyline (GENDEP) as well as in an independent, naturalistic replication sample. We then used linear discriminant analysis to calculate mRNA values cutoffs that best discriminated between responders and nonresponders after 12 weeks of antidepressants. As Macrophage Migration Inhibitory Factor and interleukin-1β might be involved in different pathways, we constructed a protein-protein interaction network by the Search Tool for the Retrieval of Interacting Genes/Proteins.

**Results:**

We identified cutoff values for the absolute mRNA measures that accurately predicted response probability on an individual basis, with positive predictive values and specificity for nonresponders of 100% in both samples (negative predictive value=82% to 85%, sensitivity=52% to 61%). Using network analysis, we identified different clusters of targets for these 2 cytokines, with Macrophage Migration Inhibitory Factor interacting predominantly with pathways involved in neurogenesis, neuroplasticity, and cell proliferation, and interleukin-1β interacting predominantly with pathways involved in the inflammasome complex, oxidative stress, and neurodegeneration.

**Conclusion:**

We believe that these data provide a clinically suitable approach to the personalization of antidepressant therapy: patients who have absolute mRNA values above the suggested cutoffs could be directed toward earlier access to more assertive antidepressant strategies, including the addition of other antidepressants or antiinflammatory drugs.

## Introduction

While there is evidence that patients with high peripheral inflammation tend to respond less to conventional antidepressants, we lack biomarkers that are reproducible across laboratories, predict response probability on an individual basis, and have clear molecular mechanisms underlying their predictive effect. The identification of biomarkers that predict treatment response is crucial in reducing the social and economic burden of depression and improving quality of life of patients. Indeed, more than one-half of patients fail to show an adequate response to first-line antidepressants (Trivedi, 2006), and one-third of patients are resistant to all available pharmacological treatments (Rush et al., 2003); therefore, there is a need to establish personalized treatment protocols that can accelerate the escalation toward adjuvant pharmacological strategies in those deemed less likely to respond. The notion of using peripheral inflammation to personalize treatment in depression is supported by a recent randomized controlled trial with the tumor necrosis factor (TNF)-α antagonist, infliximab, providing some evidence that patients with high levels of inflammation are more likely to respond to an adjuvant treatment with antiinflammatory drugs (Raison et al., 2013).

Baseline concentrations of inflammatory markers, like C Reactive Protein (CRP) and circulating cytokines, have been proposed as useful biomarkers for the identification of patients that will fail to respond to antidepressants. While depressed patients in general tend to show higher blood levels of inflammatory biomarkers compared with controls (Howren et al., 2009;Martinez et al., 2012), depressed patients who are resistant to conventional antidepressants tend to have even higher concentrations of these biomarkers, both as plasma/serum proteins (Sluzewska et al., 1997; Lanquillon et al., 2000; Fitzgerald et al., 2006; Rethorst et al., 2013) and as blood mRNA levels (Carvalho et al., 2013; Cattaneo et al., 2013; Lisi et al., 2013; Powell et al., 2013). However, despite this large amount of evidence, none of these findings have been translated yet into clinical practice, partly because different studies use different biomarkers, often measured with assays that are laboratory specific. Moreover, these assays have relative rather than absolute validity: that is, they can separate 2 groups defined as responders/nonresponders but do not necessarily predict the response probability on an individual basis. The recent study using CRP to predict differential response to nortriptyline vs escitalopram is an important step forward, because CRP is a commonly available and standardized test, but it lacks the molecular insight, since CRP is the final outcome of a number of inflammatory pathways (Uher et al., 2014).

Here we propose that the *absolute values* of Macrophage Migration Inhibitory Factor (MIF) and interleukin (IL)-1β blood mRNA molecules can be used to accurately predict antidepressant treatment response across different laboratories, because *absolute mRNA values* are more likely to be comparable independently from the laboratory setting because of the use of standard quantitation. In this study, we build on our previous work in the Genome-Based Therapeutic Drugs for Depression (GENDEP) sample, a part-randomized study with 2 active pharmacological treatment arms with nortriptyline vs escitalopram, which has been extensively described before (Uher et al., 2009, 2010; Keers et al., 2010). In our previous report (Cattaneo et al., 2013), we measured the blood mRNA *relative* expression levels of cytokines, that is, we normalized the levels of each cytokine vs the levels of internal controls (housekeeping genes). Of the many cytokines assessed (IL1α, IL-1β, IL-4, IL-6, IL-7, IL-8, IL-10, MIF, and TNF-α), only the 3 proinflammatory cytokines, IL-1β, MIF, and TNF-α, were higher in patients who later did not respond to antidepressants compared with those who did. In the present paper, we aim to: (1) select the strongest predictors (among the 3 cytokines) using multivariate logistic regression model; (2) identify the *absolute* mRNA values (number of molecules) cutoffs that best allocate individuals to the responders and nonresponders classes; (3) validate the same absolute mRNA values cutoffs in an independent sample recruited in a naturalistic setting; and (4) conduct a network analyses to assess the main targets of these proinflammatory cytokines, thus contributing to mechanistic understanding.

## Materials and Methods

### Study Design and Sample

#### GENDEP Study

The GENDEP project is an open-label, part-randomized, multicenter pharmacogenetic study with 2 active pharmacological treatment arms that has been extensively described before (Uher et al., 2009, 2014; Keers et al., 2010; Powell et al., 2012; Cattaneo et al., 2013). For the main study, 811 adults with unipolar major depression of at least moderate severity according to both the ICD–10 (World Health Organization, 1992) and the DSM–IV (American Psychiatric Association, 1994) were recruited and randomly allocated to receive flexible dosage of nortriptyline (50–150mg daily) or escitalopram (10–30mg daily) for 12 weeks. Other psychotropic medications were not allowed, with the exception of occasional use of hypnotics. Response to antidepressant medication was quantified as percentage reduction in the Montgomery–Åsberg Depression Rating Scale (MADRS) score from baseline to week 12, and responders were identified as patients with a reduction in MADRS>50%; according to this definition, approximately 55% of patients in this sample were classified as responders (Uher et al., 2009). Written informed consent was obtained from all participants, and the study was approved by the local ethics committee.

For the present study, we selected all patients who had been drug free for at least 2 weeks before entering into the trial and provided a baseline blood PaxGene tube for mRNA gene expression analysis (n=74). On average, they were in their second episode of moderately severe depression and scored, at baseline, 28.7 (±4.2) on the MADRS; according to the percent change in the MADRS score, 69% (51 of 74) were defined as responders (the selective inclusion of drug free patients may have led to a slightly more antidepressant-responsive group compared with the total sample). The main demographic and clinical features are summarized in Table 1. There were no significant differences between patients treated with escitalopram (n=38) or nortryptiline (n=36) in age (38±12.4 vs 36±9.4, *P*=.25), gender (F/M was 20/18 vs 23/13, *P*=.2), or response rate (responders/nonresponders were 26/12 vs 25/11, *P*=.6).

**Table 1.** Demographic and Clinical Information for the Two Samples of Depressed Patients

	Age	Gender (% Female)	Baseline MADRS	Responders (%)
GENDEP sample (n=74)	38.3±10.9	58.1% (31M/43F)	28.7 ±4.2	69% (51/74)
Validation sample (n=68)	39±9.5	52.9% (32M/36F)	29.5 ±3.9	66% (45/68)

#### Validation Sample

A second independent sample of n=68 depressed patients was recruited within a European multicenter collaboration on depression and analysed at the IRCCS Fatebenefratelli Brescia. The study was approved by the local Ethic Committees of the Institutes. Diagnosis of depression was confirmed by clinical interviews using the Structured Clinical Interview for DSM-IV, and severity (and treatment response) was assessed using the MADRS at baseline and at week 12. The mean (SD) MADRS score at baseline was 29.5 (±3.9); using the same criteria used in the GENDEP sample to classify responders and nonresponders, 66% (45 of 68) were defined as responders. At baseline, most patients were drug free; they then started pharmacological treatment with clinician-determined antidepressant drugs: 20 took an selective serotonin reuptake inhibitors (SSRI) (mostly escitalopram and paroxetine), 20 a serotonin and noradrenaline reuptake inhibitor (mostly duloxetine and venlafaxine), and 14 a tricyclic (mostly amitriptyline and desipramine). Participants were excluded if they were taking antipsychotics or mood stabilizers, or if they had a history of neurological or comorbid psychiatric disorders (Axis I or Axis II), substance abuse, or severe medical illness or head injury. Written informed consent was obtained from all participants, and the study was approved by the local ethics committee. Demographic and clinical features are summarized in Table 1.

### Blood Sample Collection and RNA Isolation

In both clinical groups, blood sample collection for gene expression analyses in leukocytes was performed at baseline (before starting antidepressant treatment) using PaxGene tubes. After blood collection, PaxGene tubes were kept for 2 hours at room temperature and then stored at -20°C for 24 hours and then at -80°C until their processing. RNA isolation was performed using the PaxGene Blood RNA Kit (Qiagen) according to the manufacturer’s protocols. The RNA quantity was assessed by evaluation of the A260/280 and A260/230 ratios using a Nanodrop spectrometer (NanoDrop Technologies, Wilmington, DE), and RNA quality was determined using an Agilent Bioanalyzer (Agilent Technologies). RNA samples were then stored at -80°C until their processing for gene expression analyses.

### Generation of Absolute mRNA Molecule Number through External Calibration Curves

After the initial multivariate logistic regression analysis (see Results below), which identified MIF and IL-1β as the strongest predictors of treatment response in the GENDEP sample, subsequent quantification analyses concentrated on only these 2 cytokines. We performed an *absolute* gene expression analyses to get absolute levels of cytokines mRNA expression, a novel approach that does not require normalization with “housekeeping” genes and is more likely to be comparable across different laboratories because of the use of standard quantitation. cDNA clones for human MIF and IL-1β were available from Origene (MIF NM_002415 and NM_000576, 10ug). Purified plasmid clones were then quantified using the PicoGreen method. Knowing the copy numbers and the concentration of plasmid cDNA, the precise number of molecules added to real time-PCR runs can be calculated providing a standard for specific cDNA quantification. Oligonucleotides specific for MIF and IL-1β, as well as 3 housekeeping genes (β-actin, β2-microglobulin [B2M], and GAPDH) were designed with Primer 3 software (http://www.bioinformatics.nl/cgi-bin/primer3plus/primer3plus.cgi) and used in subsequent real time reactions. Primer and probes sequences were: for β-actin, FW primer: CACACGCAGCTCATTGTAGA, RW primer: GGCATGGGTCAGAAGGATT, probe: GAAAATCTGGCACCACACCT; for B2M, FW primer ATGCCTGCCGTGTGAACCATGT, RW primer: TCAACCCTCCATGATGCTGCT, probe: CACTGAATTCACC CCCACTGA; for GAPDH, FW primer TTCACACCCATGACGAACAT, RW primer: CGAGATCCCTCCAAAATCAA, probe: GCCAAAAGGGTC ATCATCTC; for IL-1β, FW primer GGAGAATGACCTGACCACCT, RW primer: GGAGGTGGAGAGCTTTCAGT, probe: ACGATGCACC TGTACGATCA; for MIF, FW primer CGCAGAACCGCTCCTACAG, RW primer: AGGCGAAGGTGGAGTTGTTC, probe: CCGGACAGGGTCTACATCAA. For each primer and probe set, we ran a standard curve whose slope was used to calculate the PCR efficiencies, which were E_MIF_ =2.0 and E_IL-1β_=1.9. Real time PCR runs were performed in triplicate, with 1 μL of template (10ng of RNA) unknown cDNA from patients, or 1 μL of clone cDNA (8 dilution points ranging from 1e03 to 1e06), in a final volume of 2.5 μL.

The standard curve was built up with external clones containing MIF or IL-1β mRNA. Specifically, a known amount of plasmid was used to construct a calibration curve, and then unknown samples (mRNA from patients) were quantified as number of mRNA molecules (Li and Wang, 2000). After having checked for the efficiency of the assays, standard curves for MIF and IL-1β were run together (in the same reaction plate) with the mRNA samples of patients, and the threshold cycle (Ct) values of MIF or IL-1β for samples from patients were compared with the Ct values of the dilution points of the standard curve to get an absolute value of expression of each cytokine (total number of MIF or IL-1β mRNA molecules per nanogram total RNA) in each patient. In particular, a standard curve was obtained by plotting the natural log of the Ct against the natural log of the number of molecules.

To ensure that any differences in absolute levels of cytokines mRNA expression were not due to generic differences in the levels of mRNA, we measured the mRNA levels of 3 housekeeping genes in both independent samples, and we then expressed the values in term of relative expression Ratio, with “responders” as reference group.

### MIF and IL-1β Network Analysis

As MIF and IL-1β might be involved in different pathways, we constructed a protein-protein interaction network by using the Search Tool for the Retrieval of Interacting Genes/Proteins (STRING) Network Analyses software (http://string-db.org/) to identify the closest interacting genes and the main networks that are regulated.

### Statistical Analyses

Preliminary descriptive analysis, based on Mann-Whitney nonparametric test (for non-Gaussian distributions), was carried out to assess any significant differences in mRNA relative cytokines between the responder and nonresponder categories. Initially, a multiple logistic regression model was carried out in the GENDEP sample through the backward Wald method for the selection of the strongest predictors of treatment response using cytokines *relative* mRNA values of IL-1β, MIF, and TNF-α. Subsequently, univariate logistic models were performed to evaluate the associations between selected biomarkers with treatment response. The absolute values of the selected cytokines (numbers of molecules per nanogram total RNA) were then calculated, as described above. Subsequently, we used a linear discriminant analysis (LDA) to analyze the linear combination of the absolute values of the cytokines in relationship with treatment response to define a rule able to discriminate responders or nonresponders (Mc Lachlan, 2004). The resulting combination, named linear discriminant function (LDF), was then used as a linear classifier able to separate patients according to the scores of this function, indicating the probability of each individual of belonging to the responder or nonresponderclasses based on cutoff values of MIF and IL-1β mRNA molecules. Finally, to validate these findings, we assessed the performance of the same cutoff values (numbers of molecules) on generating probability scores in the second validation sample. Positive and negative predictive values (PPV, NPV), specificity, and sensitivity were calculated in both samples for the probability of being a nonresponder. Statistical analyses were carried out with SPSS 21.0 and R language and environmental v.3.0.3. (R Development Core Team, 2013). Statistical significance was set at *P*<.05.

## Results

### Baseline mRNA *relative* Expression of MIF and IL-1β, but Not TNF-α, Predicts Treatment Response in the GENDEP Sample

As mentioned above, we had previously found that nonresponders in the GENDEP sample had higher baseline mRNA *relative* expression levels of 3 proinflammatory cytokines, IL-1β, MIF, and TNF-α, compared with responders; these findings were present for both escitalopram and nortryptiline nonresponders (Cattaneo et al., 2013). In those analyses, we did not apply any variable selection procedure; however, we found that TNF-α had the lowest predicting power (adjusted R^2^=0.19) compared with both IL-1β (adjusted R^2^=0.31) and MIF (adjusted R^2^=0.37) (Cattaneo et al., 2013).

In the present study, we detected the biomarkers that were more strongly associated with treatment response by using a multiple logistic regression model applying the backward Wald method for the variable selection, which automatically removes variables that do not add more significance to the model. We found that MIF and IL-1β were strongly associated with treatment response: the odd ratios (ORs) of being a nonresponder vs being a responder (obtained by univariate logistic models) were OR=1.32 (95% CI 1.04–1.70, *P*=.034) for MIF, and OR=1.24 (95% CI 1.02–1.54, *P*=.049) for IL-1β, with higher levels predicting lack of response. Moreover, the power of the model increased (with an R2 of Negelkerke index increasing from 0.86 to 0.95) when IL-1β was evaluated together with MIF in the same logistic regression model (OR=1.44, *P*=.030). TNF-α, in contrast, did not significantly increase the predictive power (*P*>.05) and was removed from the model by backward selection method. Of note, the ORs were similar when we analyzed separately the response prediction to escitalopram (OR=1.34, *P*=.029 for MIF, and OR=1.29, *P*=.035 for IL-1β) and to nortriptyline (OR= 1.35, *P*=.038 for MIF, and OR=1.20, *P*=.045 for IL-1β), indicating that these cytokines predict treatment response to both antidepressants.

### Baseline mRNA *Absolute* Expression of MIF and IL-1β Accurately Discriminate between Responders and Nonresponders at the Individual Level

We measured the absolute blood mRNA expression of MIF and IL-1β in the GENDEP sample in order to get *absolute* (and not relative) mRNA measurements. Overall, means±SEM in the whole sample were 66.8±3.5 x10^6^ for IL-1β and 130.2±5.2 x10^6^ for MIF. Consistently with our previous findings using *relative* gene expression approach (Cattaneo et al., 2013), we found that the mean absolute number of molecules for MIF and IL-1β were significantly higher in nonresponders compared with responders (mean± SEM; IL-1β: 83.1±4.8 x10^6^ in nonresponders vs 50.4±2.1 x10^6^ in responders, t=11.7, DF=72, *P*<.001; MIF: 102.5 x10^6^±4.2 in nonresponders vs 55.4 x10^6^±1.9 in responders, t=7.2, DF=72, *P*<.001).

We then used an LDA to analyze the linear combination of the absolute values of MIF and IL-1β in relationship with treatment response to define a rule able to discriminate between responders and nonresponders (Mc Lachlan, 2004). Specifically, we first used LDA to calculate the coefficients for each biomarker (resulting in 0.056 for MIF and 0.036 for IL-1β, respectively) and then used the LDF to calculate the probability of each individual of belonging to the responder or nonresponder classes based on cutoff values of MIF and IL-1β mRNA molecules (Table 2). The table shows the relative probability of being a responder (in green) or nonresponder (in red) based on the LDF and according to the quantitative chosen cutoff values. The top-left cells represent patients with the lowest levels of inflammation and the highest probability of being a responder, virtually equal to 100%; with increasing levels of either or both cytokines, the probability of being a responder decreases dramatically, with bottom-right cells being characterized by a probability of being a responder virtually equal to zero. Specifically, green (responder) indicates >73% probability of being a responder and <27% probability of being a nonresponder; red (nonresponder) indicates <32% probability of being a responder and >72% probability of being a nonresponder; and orange indicates intermediate values. Based on these criteria, this sample had n=35 (47%) of subjects classified as responders (MIF≤60x10^4^ and IL-1β≤85x10^4^), n=23 (31%) of subjects classified as nonresponders (MIF>95x10^4^ and IL-1β>50x10^4^), and n=16 (22%) of subjects classified as intermediates (MIF=60x10^4^-95x10^4^ and IL-1β=50x10^4^-85x10^4^).

**Table 2.** Cutoff Values of MIF and IL-1β Molecules and Probability Scores of Being a Responder or a Nonresponder in the First Sample of Depressed Patients (GENDEP Sample)

Number of molecules (IL-1β or MIF) per nanogram total RNA	**IL-1β ≤ 50x10** ^**4**^	**50 x10** ^**4**^ **< IL-1β ≤ 85 x10** ^**4**^	**IL-1β > 85 x10** ^**4**^
MIF ≤ 60x10^4^	Responder probability >0.995	Responder probability >0.73	Responder probability >0.37
	Nonresponder probability <0.005	Nonresponder probability <0.27	Nonresponder probability <0.63
60 x10^4^< MIF ≤ 95 x10^4^	Responder probability >0.32	Responder probability 0.008–0.995	Responder probability <0.73
	Nonresponder probability <0.68	Nonresponder probability 0.005–0.99	Nonresponder probability >0.27
MIF > 95x10^4^	Responder probability 0.03–0.93	Responder probability <0.32	Responder probability <0.008
	Nonresponder probability 0.07–0.97	Nonresponder probability >0.72	Nonresponder probability >0.99

White columns on the right represent MIF cutoffs (in term of number of molecules) and white rows on the top represent IL-1β cutoffs (in term of number of molecules); each cells or in green, orange, or red indicates the probability of being a responder or a nonresponder: green (responder) indicates >73% probability of being a responder and <27% probability of being a nonresponder; red (nonresponder) indicates <32% probability of being a responder and >72% probability of being a nonresponder; and orange indicate intermediate values.

We then calculated PPV, NPV, sensitivity, and specificity for being a nonresponder using the specific cutoff values (MIF>95x10^4^ and IL-1β>50x10^4^; red cells in the table). 100% of those classified as nonresponders (n=14) were true nonresponders (PPV=100%), and all true responders (n=51) were identified as responders (specificity=100%). This is ideal in terms of clinical practice: using this approach, only true nonresponder patients would be exposed to a more rapid or assertive antidepressant strategies (see also Discussion). Nine (39%) of nonresponders were not identified as such (NPV=85%, sensitivity=61%): in terms of clinical practice, they would continue to receive standard treatment.

We found no difference in the expression levels of the 3 housekeeping genes in this sample; mean±SEM in responders vs nonresponders were: β-actin: 1.08±0.7 vs 1.09±0.6; B2M: 1.11±0.8 vs 1.11±0.4; GAPDH: 1.03±0.4 vs 1.09±0.6 (all *P*>.05).

### MIF and IL-1β mRNA Molecules as Predictors of Treatment Response Are Validated in an Independent Sample

We also measured the number of MIF and IL-1β mRNA molecules in the second independent sample of 68 depressed patients. Overall, the absolute RNA levels observed in this second validation sample were similar to those observed in the first group (mean and SEM were 66.5±3.4 x10^6^ for IL-1β and 74.8±2.9 x10^6^ for MIF). As a first validation, we found that, similarly to the GENDEP sample, nonresponders had higher numbers of MIF and IL-1β molecules compared with responders (mean±SEM; IL-1β: 78.5±3.5x10^6^ in nonresponders vs 54.4±3.2x10^6^ in responders, t=9.8, DF=50, *P*<.01; MIF: 94.3±3.2x10^6^ in nonresponders vs 55.3±2.7x10^6^ in responders, t=8.2, DF=50, *P*<.001). The results remain the same also when analyzing separately responders and nonresponders to SSRI (*P*=.008 for IL-1β and *P*=.009 for MIF) or SNRI (*P*=.003 for IL-1β and *P*=.009 for MIF), replicating the evidence from the GENDEP sample that these cytokines predict treatment response to both antidepressants.

As reported in Table 3, we then used the second sample to validate our predictive model based on the absolute mRNA cutoff values identified in the first sample. Indeed, we obtained probability scores for being responders/non-responders that were very similar to those obtained in the first sample (*P*>.05 for all comparisons between probability scores), again, with top-left cells representing patients with the lowest levels of inflammation and the highest probability of being a responder, and bottom-right cells representing those with the highest levels of inflammation and the lowest probability of being a responder. Specifically, green (responder) indicates >82% probability of being a responder and <18% probability of being a nonresponder; red (nonresponder) indicates <28% probability of being a responder and >72% probability of being a nonresponder; and orange indicate intermediate values. Based on these criteria, this sample had n= 29 (43%) of subjects classified as responders (MIF ≤60x10^4^ and IL-1β≤85x10^4^), n= 13 (19%) of subjects classified as non-responders (that is, with MIF>95x10^4^ and IL-1β>50x10^4^), and n=26 (38%) of subjects classified as intermediates (that is, MIF=60x10^4^-95x10^4^ and IL-1β=50x10^4^-85x10^4^). Similar to the GENDEP sample, 100% of those classified as nonresponders (n=13) were true nonresponders (PPV=100%), and all true responders (n=45) were identified as responders (specificity=100%). Ten (43%) of nonresponders were not identified as such (NPV=82%, sensitivity=56%).

**Table 3.** Cutoff Values of MIF and IL-1β Molecules and Probability Scores of Being a Responder or a Nonresponder in the Second Independent Sample of Depressed Patients

Number of Molecules (IL-1β or MIF)/ng Total RNA	IL-1β ≤ 50x10^4^	50 x10^4^ < IL-1β ≤ 85 x10^4^	IL-1β > 85 x10^4^
MIF ≤ 60x10^4^	Responder probability >0.99	Responder probability >0.82	Responder probability >0.39
	Nonresponder probability <0.001	Nonresponder probability <0.18	Nonresponder probability <0.61
60 x10^4^< MIF ≤ 95 x10^4^	Responder probability >0.28	Responder probability 0.001–0.99	Responder probability <0.82
	Nonresponder probability <0.72	Nonresponder probability 0.001–0.99	Nonresponder probability >0.18
MIF > 95x10^4^	Responder probability 0.007–0.97	Responder probability <0.28	Responder probability <0.001
	Nonresponder probability 0.03–0.99	Nonresponder probability >0.72	Nonresponder probability >0.99

White columns on the right represent MIF cutoffs (in term of number of molecules) and white rows on the top represent IL-1β cutoffs (in term of number of molecules); each cells or in green, orange, or red indicates the probability of being a responder or a nonresponder: green (responder) indicates >82% probability of being a responder and <18% probability of being a nonresponder; red (nonresponder) indicates <28%; probability of being a responder and >72% probability of being a nonresponder; and orange indicate intermediate values.

Again, we found no difference in the expression levels of the three housekeeping genes in this sample; mean ±SEM in responders vs nonresponders were: β-actin: 1.09±0.8 vs 1.10±0.7; B2M: 1.08±0.5 vs 1.10±0.7; GAPDH: 1.06±0.5 vs. 1.08±0.7 (all *P* > .05).

### MIF and IL-1β Network Analysis Identifies Different Molecular Targets

**Figure 1.**
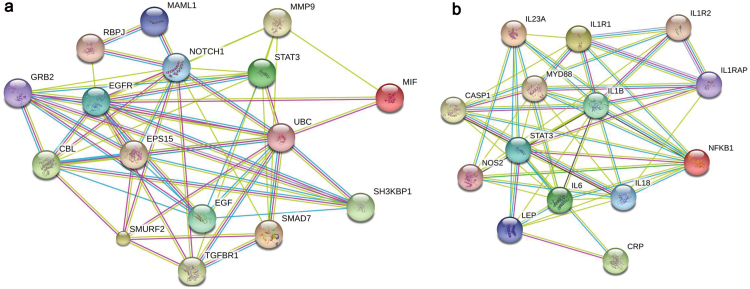
Representative interaction between Macrophage Migration Inhibitory Factor (MIF) (Figure 1a) or interleukin (IL)-1β (Figure 1b) and their neighbors’ targets, where nodes (genes) can be either colored (if they are directly linked to the input, in that case MIF) or white (nodes of a higher interaction/depth-this is not the case). Lines represent predicted functional edges of interaction between nodes, and they are represented with eight different colors according to the type of evidence and the predictive method used (neighborhood, gene fusion, co-occurrence, coexpression, experiments, databases, and textmining): green line,activation; red line, inhibition; blue line, binding; light blue line, phenotype; violet, catalyzes; pink, posttranslational mechanism; black, reaction; yellow, coexpression (http://string-db.org).

The network analysis showed that MIF and IL-1β were directly linked as reciprocal targets (data not shown), indicating that increased activation of one cytokine has downstream effects also on the other. However, the 2 cytokines connected to different and specific clusters of targets. As shown in Figure 1a, MIF did not show any interactions with other proinflammatory cytokines, and it interacted mainly with ubiquitin C via matrix metalloproteinase 9, which, in turn, is involved in the regulation of genes known to have an effect on neurogenesis, neuroplasticity, and cell proliferation, like Endothelial Growth Factor, Notch, and SMAD proteins (Anacker et al., 2013; Marschallinger et al., 2014). On the contrary, the neighbor targets of IL-1β (Figure 1b) were mainly represented by proteins with inflammatory properties, including IL-6 and CRP, and by other molecules involved in the upstream or downstream regulation of the inflammatory signal, like Toll Like Receptors, Caspases (CASP), and nuclear factor kappa-light-chain-enhancer of activated B cells; interestingly all of these partners are related to the inflammasome complex or are mediators of oxidative stress, and they are well known to cause neurodegenerative effects (Leemans et al., 2011; Radi et al., 2014).

## Discussion

In this study we report that the *absolute* numbers of MIF and IL-1β mRNA molecules are both accurate and reliable predictors of antidepressant response, identifying, for the first time, an mRNA-based biomarker approach that is independent from local experimental settings and does not require “relative” quantification using housekeeping genes. Of note, the predictive power of these proinflammatory cytokines, and in particular the absolute values of mRNA molecules identified as the best cutoffs for prediction of nonresponse on an individual basis, were independently replicated in two clinical samples: a randomized trial that we had previously used to measure relative mRNA expression and to identify the top mRNA transcripts of interest (Cattaneo et al., 2013); and a naturalistic cohort that has been independently recruited for this study. Both samples demonstrate identical PPV and specificity of 100% for nonresponders. This is the ideal scenario in clinical practice: patients with mRNA numbers above the suggested cutoffs (“red” in Tables 2 and 3) could be directed toward early access to more assertive antidepressant strategies, including augmentation with other antidepressants or antiinflammatory drugs, while those with mRNA numbers below the suggested cutoffs could be directed toward standard care. Most importantly, this would allow a therapeutically conservative approach, where no true responders would be exposed unnecessarily to more assertive pharmacological strategies.

Depressed patients who are resistant to conventional antidepressants have higher concentrations of inflammatory biomarkers in plasma or serum (Sluzewska et al., 1997; Lanquillon et al., 2000; Fitzgerald et al., 2006), and successful antidepressant treatment has been associated with a reduction in the levels of proinflammatory cytokines (Abdel-Salam et al., 2003; Roumestan et al., 2007; Cattaneo et al., 2013). As mentioned above, we conducted the first study showing that (relative) mRNA expression levels of IL-1β, MIF, and TNF-α are higher in nonresponders. Our findings have been corroborated by Belzeaux and colleagues (2012), who identified a combination of mRNA signatures of TNFα and IL-1β as best predictors of antidepressant response in a case study, and by Powell and colleagues (2013) in a separate study of a different GENDEP sample, who investigated the mRNA expression of 84 genes related to inflammation and found that nonresponders have significantly higher baseline mRNA levels of TNF and other TNF-target genes. Cytokine mRNA levels may also be involved in the genetic regulation of treatment response: for example, Uher et al. (2010) have found, in yet another GENDEP sample, a single nucleotide polymorphism (rs1126757) in the IL-11 gene with suggestive genome-wide significance, and Powell et al. (2013) have found that only carriers of the IL-11 A allele (who have a better response) show a significant reduction in IL-11 mRNA expression following citalopram. Together with the present findings showing accurate and reliable prediction of antidepressant response by *absolute* mRNA levels in 2 independent samples, all these studies show that peripheral blood mRNA analyses are one of the most promising approaches for biomarker discovery in mental health (Hepgul et al., 2013). This notion is supported by the fact that about 80% of genes are coexpressed, and similarly modulated as mRNA levels, in peripheral blood cells and brain tissues (Sullivan et al., 2006). Moreover, molecular mechanisms activated by early life stress, a well-known risk factor for antidepressant nonresponse (Nanni et al., 2012), produce similar epigenetic changes in human blood cells and human neuronal cells, leading to long-term dysregulation of the stress hormone system and global changes in the function of immune cells as well as of brain areas associated with stress regulation (Klengel et al., 2013; Uher et al., 2014).

In our study, we find that MIF and IL-1β mRNA levels predict treatment response across antidepressants classes, that is, for both escitalopram and nortriptyline in the GENDEP samples as well as for both SSRIs and SNRIs in the naturalistic cohort. These findings may be perceived as in contrast with the recent paper by Uher et al. (2014) showing (in a larger, different GENDEP sample) that high levels of serum CRP predicted lack of treatment response to the SSRI, escitalopram, but not to the tricyclic (and noradrenergic uptake inhibitor) nortriptyline. The authors explain this by speculating that antidepressants with a noradrenergic action may have antiinflammatory properties (Uher et al., 2014), and, in fact, we and others have found that noradrenergic or SNRI antidepressants have antiinflammatory properties in vitro (Horowitz et al., 2014). However, the antiinflammatory properties of SSRI drugs are also well known (Tynan et al., 2012; Al-Amin et al., 2013; Cattaneo et al., 2013). Our network analysis may shed some light on this apparent discrepancy, as CRP is only loosely connected with IL-1β (and it is not connected with MIF), indicating that subjects with high levels of CRP may be different from those with high levels of MIF or IL-1β; indeed CRP levels in the aforementioned paper predicted only around 11% of MADRS-based response, as opposed to 40% to 50% of the variance explained by cytokines mRNA levels in our study (Cattaneo et al., 2013). Moreover, because MIF and IL-1β are “neighbor targets,” it is possible that having high levels of either of these cytokines (i.e., above the top cutoff) is enough to activate downstream targets of both, thus affecting both the neurogenesis/neuroplasticity targets of MIF and the inflammasone/neurodegeneration targets of IL-1β. These widespread molecular abnormalities would likely inhibit the response to a wide range of antidepressants. Of note, taken together with the other GENDEP studies mentioned above by Uher et al. (2010) and Powell et al. (2013), our study and the study by Uher et al. (2014) all point to a prominent role of increased inflammation in the lack of antidepressant response in the GENDEP cohort. However, as these studies have been conducted in different and only partially overlapping samples, it is difficult to determine if and how these genetic, serum, and mRNA biomarker cluster together.

High levels of inflammation can prevent response to antidepressants, because it can interfere with the same biological processes that are crucial for antidepressant therapeutic action. For example, high levels of inflammation increase the expression and activation of monoamine transporters, reduce tryptophan availability, inhibit neuropeptide and growth factors involved in neuroplasticity, and interfere with the kynurenine pathways, leading in turn to a reduction in neurogenesis and glutamate dysfunction (Zunszain et al., 2012; Felger and Lotrich, 2013). Of note, inflammation can be triggered by childhood trauma (Danese et al., 2007, 2008; Hepgul et al., 2013; Baumeister et al., 2015), which is in itself associated with poor antidepressant response (Nanni et al., 2012); thus, it is possible to speculate that a cluster of individuals have both a history of childhood trauma and increased inflammation, eventually leading to lack of antidepressant response (Nemeroff et al., 2003; Nanni et al., 2012; Coelho et al., 2014). However, it is also known that genetic variability in inflammatory genes, such as IL-6, IL-11, and TNF-α, as well as in enzymes involved in the prostaglandins and kynurenine pathways, contributes to both the risk of depression and to antidepressant response (Bull et al., 2009; Dowlati et al., 2010; Uher et al., 2010; Bufalino et al., 2013), thus suggesting that a different cluster of individuals expresses high levels of inflammation and lack of treatment response through a predominantly genetic path. Indeed, it is possible that the better accuracy and validity of mRNA gene expression as biomarkers of treatment response are due to the ability of these molecules to integrate genomic variability and environmental effects. Importantly, inflammatory genes could represent not only biomarkers predicting lack of antidepressant response but also novel targets for antidepressant therapies, and indeed clinical trial have been conducted to test the efficacy of antiinflammatory drugs. Müller et al. (2006) was the first to conduct a randomized controlled clinical trial to evaluate the efficacy of celecoxib added to antidepressant drugs and reported improved antidepressant effects. Subsequent studies confirmed these findings (Akhondzadeh et al., 2009; Hashemian et al., 2011; Abbasi et al., 2012). Moreover, Raison and colleagues (2013) have recently reported that a specific antagonist of TNFα improves depressive symptoms in patients with high inflammation at baseline. No clinical trials have been conducted with drugs able to inhibit MIF or IL-1β; however, there are promising data in animals that IL-1β receptor antagonism prevents long-term cognitive impairment and memory deficits, which are clinical features observed in depressed patients (Barichello et al., 2015).

The targets identified by the network analyses of MIF and IL-1β shed some interesting light on the putative molecular mechanisms underlying their predictive effects. MIF promotes the expression and function of multiple cytokines and chemokines, including TNF-alpha, IL-6, CXCL1, and CCL2 (Gregory et al., 2006; Toh et al., 2006; Fan et al., 2011; Santos et al., 2011). However, MIF also plays a role as a physiological counter-regulator of glucocorticoid antiinflammatory action (Leech et al., 2000; Aeberli et al., 2006) by regulating glucocorticoid-induced leucine zipper, which in turn not only has antiinflammatory functions but also is involved in the effects of stress on neurogenesis (Anacker et al., 2013). Finally, MIF also interacts with genes involved in neurogenesis, neuroplasticity, and cell proliferation, like Endothelial Growth Factor, Notch, and SMAD proteins (Anacker et al., 2013; Marschallinger et al., 2014). Indeed, MIF has been shown to promote neuroplasticity and neuroprotective processes under physiological conditions, but it can also increase the production of proinflammatory cytokines under conditions of stress. Moreover, MIF is modulated by glucocorticoids, and high, antiinflammatory doses of glucocorticoids inhibit MIF secretion; however, during pathological conditions characterized by glucocorticoid resistance, such as depression, it is conceivable that levels of MIF are increased (Bloom et al., 2014). In contrast, IL-1β contributes to the activation of other cytokines (IL-18, IL-6) and mediators of oxidative stress, like CASP1, CASP9, and Nitric oxide synthase 2, related to the inflammasome complex, also comprising the Nod-like Receptor, the precursor pro-caspase-1, and the adaptor ASC. Interestingly an overactivation of the inflammasome in peripheral blood cells of depressed patients has been recently demonstrated, providing support to its putative role in the pathogenesis of depression and its treatment (Alcocer-Gomez and Cordero, 2014). We would advocate that any of these pathways might represent a target for novel antidepressant therapies.

The finding that markers of peripheral inflammation can predict the response to an antidepressant can be interpreted as an example of a peripheral correlate of central (immune) alterations. Indeed, increased neuro-inflammation has been reported in the brain of patients with depression; for example, postmortem studies have found an increased proportion of primed microglia and higher mRNA levels of several chemokines involved in the recruitment of monocytes (Torres-Platas et al., 2014); and a recent neuroimaging study has found increased microglia activation (translocator protein density) using positron emission tomography (Setiawan, et al., 2015). Indeed, it has recently been proposed that microglial activation in psychiatric disorders could not only contribute to the brain pathology but also influence treatment response (Reus et al., 2015). It is also, however, important to highlight that readily available, blood-based peripheral biomarkers are relevant in psychiatry even if they do not directly reflect brain-based mechanisms when they reliably predict important clinical features such as risk, vulnerability, outcome, and treatment response.

The findings of the present study need to be interpreted in the light of important limitations. First, although we have validated our results in 2 independent samples, the sample size is relatively small, and therefore these findings should be replicated in larger datasets. Indeed, we have assessed “response” rather than “remission,” as the latter would have led to even smaller groups available for the analyses. Second, the identified biomarkers predict the response to antidepressants in general and are not specific to one or the other of the antidepressant classes; therefore, they cannot be used to guide the choice of antidepressants but rather to identify patients that may benefit from an early access to adjuvant therapies. Third, these findings may be relevant only to patients that, as in our samples, show no historical evidence of chronicity or of treatment resistance to multiple antidepressants, although we would argue that this is indeed the kind of patients that would benefit most from our proposed personalized approach of early access to assertive antidepressant strategies based on the biomarkers profile.

In summary, here we provide evidence that the measurements of the number of MIF and IL-1β mRNA molecules, which is an absolute and thus reliable quantitation that could be adopted across laboratories, may help the clinicians in the prediction of antidepressant response, so that patients with mRNA numbers above the suggested cutoffs (“red”) could be directed toward early access to more rapid escalation of assertive antidepressant strategies. However, randomized controlled trials testing the use of biomarkers vs treatment as usual need to be conducted in order to deliver clear guidelines, especially considering the increased risk of adverse effects when combining conventional antidepressants with antiinflammatories (Andrade et al., 2010).

## Statement of Interest

Professor Pariante has received research funding from Johnson & Johnson as part of a programme of research on depression and inflammation, and speaker’s fee from Lundbeck. In addition, Professor Pariante has received research funding from the Medical Research Council (UK) and the Wellcome Trust for research on depression and inflammation as part of two large consortia that also include Johnson & Johnson, GSK, Pfizer and Lundbeck.
